# Adhesion and Hydrothermal Performance of Epoxy–Dicyandiamide Adhesive Film for PMMA-PETR

**DOI:** 10.3390/polym18151918

**Published:** 2026-08-05

**Authors:** Guoliang Yu, Lei Wang, Yue Li, Hu Lyu, Dongzhou Sun, Wei Dong, Shudi Liu, Yanting Du, Kexin Ning, Dawei Zhang, Zhiqiang Ning, Xianzhi Kong

**Affiliations:** 1 Institute of Petrochemistry, Heilongjiang Academy of Sciences, Harbin 150040, China; yuguoliang@hipc.org.cn (G.Y.); liyue@hipc.org.cn (Y.L.); haitun@hipc.org.cn (H.L.); sundongzhou@hipc.org.cn (D.S.); dongweivvv@163.com (W.D.); liushudi@hipc.org.cn (S.L.); duyanting1206@163.com (Y.D.); 2 AVIC Shenyang Aircraft Design and Research Institute, Shenyang 110035, China; 13998278321@139.com; 3 State Key Laboratory of Woody Oil Resources Utilization, Northeast Forestry University, Harbin 150040, China; ningkexin2023@outlook.com (K.N.); zhangdawei@nefu.edu.cn (D.Z.)

**Keywords:** imidazole/alkanolamine composite accelerator, epoxy/dicyandiamide adhesive film, curing process, hydrothermal aging performance

## Abstract

Epoxy/dicyandiamide adhesive film is required for the bonding of polymethyl methacrylate (PMMA) aircraft cockpit edges. An imidazole/alkanolamine composite accelerator was developed to satisfy the requirement of maintaining a bonding temperature below 90 °C for this structure, successfully reducing the curing temperature of the adhesive film from 180 °C to 85 °C. The curing process of the low-temperature curing adhesive film and its hydrothermal aging resistance after curing were investigated using mechanical testing, Fourier transform infrared spectroscopy (FTIR), dynamic mechanical analysis (DMA), and thermogravimetric analysis (TGA). The results indicate that the curing process of 85 °C for 6 h enables the adhesive film to fulfill the adhesion strength requirements for aircraft cockpit edge applications. The cured adhesive film is found to exhibit limited intrinsic water resistance due to its low crosslinking density. Therefore, external edge sealing is required in practical engineering applications to isolate moisture. This work provides an application-specific one-component epoxy/dicyandiamide adhesive film for the low-temperature flexible bonding of aviation PMMA cockpit edges to PETR, and clarifies its adhesion performance and hydrothermal aging behavior under the target service scenario.

## 1. Introduction

Epoxy/dicyandiamide (DICY) adhesive film is a one-component thermosetting adhesive formulated with a linear epoxy resin matrix, a dicyandiamide curing agent, and functional additives including reinforcing fillers, curing accelerators, and antioxidants/UV absorbers, with a curing temperature exceeding 180 °C [1]. This system has been widely utilized in temperature-resistant fields such as aerospace, transportation, electronic packaging, and composite materials, due to its excellent adhesion strength, corrosion resistance, thermal stability, and favorable storage and processing characteristics [2,3]. However, the widespread application of the epoxy/dicyandiamide system in medium-temperature (≤100 °C) bonding fields is restricted by its excessively high curing temperature.

**Figure 1:**
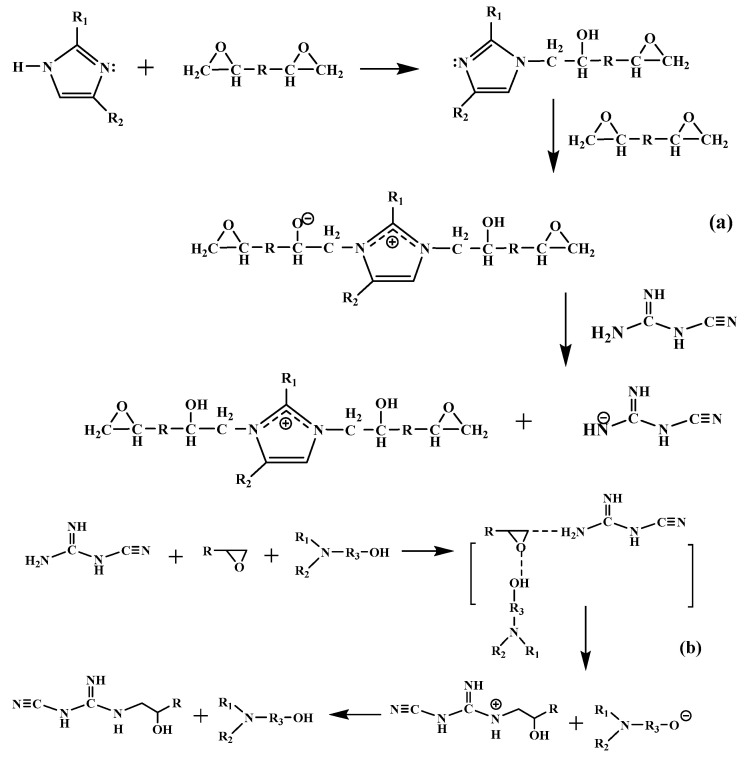
The mechanism of (**a**) imidazole accelerators [5]; (**b**) alkanolamine accelerators [6] on epoxy/dicyandiamide systems.

A significant reduction in the curing temperature, along with the enhancement in other properties of the cured sample, can be achieved through the incorporation of an appropriate amount of accelerators into the system by lowering the reaction activation energy while the pot life is maintained. This area has been recognized as a major research focus in the modification of medium-to-high-temperature curing epoxy resins in recent years. Among these, imidazole and alkanolamine accelerators have been extensively investigated due to their favorable compatibility with epoxy resins and pronounced accelerating efficiency [4]. The acceleration mechanism of an imidazole accelerator on the epoxy/dicyandiamide is illustrated in Figure 1a, and the alkanolamine acceleration mechanism of an accelerator is shown in Figure 1b [5,6].

Specifically, an alkoxy intermediate is initially formed via the reaction between the secondary amine of the imidazole accelerator and the epoxy group. Meanwhile, an alkoxy anion is generated through the reaction of the tertiary amine with the epoxy group, followed by the abstraction of a proton from dicyandiamide to achieve saturation, whereby a dicyandiamide anion is formed. The reactivity of this anion toward epoxy groups is significantly higher than that of dicyandiamide itself. Within the epoxy/dicyandiamide system, nucleophilic promotion and intermolecular activation are primarily driven by triisopropanolamine. An active ion-pair intermediate is generated via the nucleophilic attack of its tertiary amine nitrogen atom on the cyano group of dicyandiamide. Simultaneously, a hydrogen bond is formed between the hydroxyl group and the epoxy group, whereby localized solubilization and electron cloud polarization are achieved. The activation energy of the ring-opening reaction is thus lowered, enabling the rapid curing of the latent system under medium-to-low-temperature conditions [7,8].

The heat deflection temperature of the aviation PMMA substrate utilized for windshield cockpit edge flexible connections is below 100 °C. Although favorable processing characteristics are imparted to epoxy adhesives by dicyandiamide-type latent curing agents, a curing temperature exceeding 180 °C is typically required for such systems. The incorporation of various latent accelerators into the system has been adopted as the mainstream strategy for the reduction in the curing temperature. For instance, the reaction activation energy of the system can be effectively reduced by substituted urea compounds [1]. Recent studies have also shown that adhesive systems designed for thermally sensitive substrates can be cured below 100 °C, preferably around 80 °C [9]. Nevertheless, for the aviation PMMA-PETR cockpit edge flexible bonding system investigated in this work, the curing temperature is required to be controlled below the thermal limitation of PMMA while maintaining sufficient bonding and peel performance. High nucleophilic activity is exhibited by imidazole compounds; however, it has been indicated by relevant kinetic studies that premature vitrification is easily induced within the system during isothermal curing at lower temperatures if imidazole is utilized individually. Consequently, the reaction control mechanism is transformed from chemical control to diffusion control, whereby the final conversion rate is restricted and network crosslinking remains incomplete [10]. Furthermore, although auxiliary catalysis can be provided by tertiary amine compounds such as alkanolamines through the formation of hydrogen bonds via hydroxyl groups, the initial reaction temperature of dicyandiamide can hardly be reduced to below 100 °C when these compounds are used individually [6].

The edges of the PMMA cockpit are bonded to the polyester ribbon (PETR) via the epoxy/dicyandiamide adhesive film, thereby establishing the PMMA-AF-PETR structure.

This bonded PMMA-AF-PETR structure is subjected to erosion by moisture, heat, and their synergetic effects during service. Plasticization of the polymer matrix is induced by water molecules; upon penetration into the crosslinked network, hydrogen bonds are formed between these water molecules and the polar groups of the epoxy molecular chains. Consequently, the original intermolecular interactions are replaced, and the free volume of the molecular chains is increased. Microscopically, the mobility of the molecular segments is enhanced, while macroscopically, a reduction in the glass transition temperature (Tg) of the matrix and decreases in the modulus and strength of the material are induced. The structural equilibrium of the matrix is also disrupted by water penetration, whereby structural relaxation is triggered and property gradients are formed. This phenomenon has been recognized as one of the primary inducements for the rapid degradation of mechanical properties in highly polar epoxy systems during the initial stage of hydrothermal exposure [10,11,12].

Furthermore, it has been indicated that although plasticization is generally characterized by reversibility, an irreversible chemical degradation process is frequently accompanied under extreme hydrothermal conditions. Among these, phenomena such as hydrolysis [10,11,12,13,14], post-curing [15], degradation [10,11,12,13,16,17,18], and structural relaxation [10] are identified as the key factors governing the structural mechanical properties and durability of polymer materials [10,11,12,13,14,15,16]. Particularly for low-temperature curing systems, the adhesive bulk is typically more susceptible to water molecules, whereby network hydrolysis is accelerated and the deterioration in mechanical properties is induced, owing to the relatively low crosslinking density inherent to such systems. Consequently, the degradation behavior of this flexible connection structure can be clarified and a theoretical foundation for the mandatory external sealing protection in practical engineering can be provided through the investigation of its hydrothermal aging mechanism, whereby the safe service of the aircraft cockpit is guaranteed.

The novelty of this study lies in the development of an application-specific one-component epoxy/dicyandiamide adhesive film for the flexible bonding of aviation PMMA cockpit edges to PETR. Unlike two-component adhesives that require on-site mixing, the present adhesive film can be directly applied, thereby improving processing convenience and reducing assembly uncertainty. Although low-temperature curing adhesives have been reported, the present work focuses on a PMMA-PETR flexible connection in which the curing temperature is required to be controlled below the thermal limitation of aviation PMMA. In this study, an epoxy/dicyandiamide system was employed as the adhesive matrix, and an imidazole/alkanolamine combination was utilized as the composite accelerator. The effects of the curing temperature and curing time on the adhesion performance were investigated, and the chemical structure, thermomechanical properties, and thermal decomposition behavior of the cured adhesive films were characterized by mechanical testing, Fourier transform infrared spectroscopy (FTIR), dynamic mechanical analysis (DMA), and thermogravimetric analysis (TGA). Hydrothermal aging of the PMMA-AF-PETR bonded joint was conducted under environmental conditions for aviation material qualification, namely 80 °C and 90% relative humidity, and the subsequent variations in adhesion performance and chemical structure during aging were evaluated through mechanical testing, FTIR, and TGA. Regarding the design criteria for the windshield cockpit edge flexible connection, the required adhesion properties are specified as a bonding strength of ≥200.00 kN/m and a 90° peel strength of ≥23 N/cm at room temperature, and a bonding strength of ≥100.00 kN/m and a 90° peel strength of ≥23 N/cm at 100 °C. Meanwhile, the processing window is restricted to a bonding temperature below 90 °C and a curing time below 8 h.

## 2. Materials and Methods

### 2.1. Main Materials

**Figure 2:**
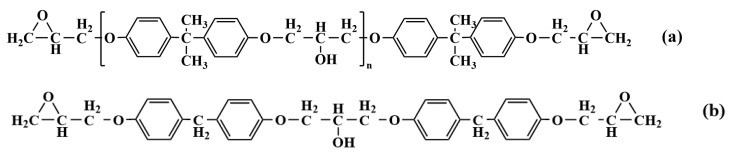
Epoxy resin structural formulas: (**a**) E-21 epoxy resin; (**b**) NPEF-170 epoxy resin.

E-21 epoxy resin (bisphenol A type, Figure 2a), China Bluestar (Group) Co., Ltd. (Beijing, China); NPEF-170 epoxy resin (bisphenol F type, Figure 2b), Nan Ya Epoxy Resin (Kunshan) Co., Ltd. (Kunshan, Jiangsu, China); aviation PMMA, Arkema (Colombes, France); PETR, Bally Ribbon Mills (BRM, Bally, PA, USA); 2-ethyl-4-methylimidazole (2E4MI), purity 99%, Shanghai Macklin Biochemical Technology Co., Ltd. (Shanghai, China); 1-methylimidazole (DY070), purity 99%, Shanghai Macklin Biochemical Technology Co., Ltd. (Shanghai, China); triisopropanolamine (TIPA), purity 95%, Shanghai Macklin Biochemical Technology Co., Ltd. (Shanghai, China); 2,4,6-tris(dimethylaminomethyl)phenol (DMP-30), purity 95%, Shanghai Macklin Biochemical Technology Co., Ltd. (Shanghai, China). All materials were directly used without further purification.

### 2.2. Experimental Methods

#### 2.2.1. Preparation of Epoxy/Dicyandiamide Adhesive Film

Epoxy resin, dicyandiamide, and the imidazole and alkanolamine accelerators were sequentially added to an internal mixer and kneaded at 40 °C for 120 min. Therein, E-21 epoxy resin and NPEF-170 epoxy resin were charged at a mass ratio of 2:1, and the equivalent ratio of epoxy groups to active amine hydrogens (from dicyandiamide) was maintained at 1:0.6. The composite accelerator was incorporated at a dosage of 1.5 wt% relative to the total mass of the epoxy resin and dicyandiamide, and the molar ratio of 2E4MI:DY070:DMP-30:TIPA in the composite accelerator was set to 9:1:18:2. The selection of the accelerator components was based on previous studies concerning tertiary amine and imidazole accelerators in epoxy curing systems. Yang et al. reported that tertiary amine accelerators, especially DMP-30, can significantly reduce the apparent activation energy of epoxy curing reactions [19]. Liu et al. further demonstrated the synergistic accelerating effect between DMP-30 and an imidazole accelerator in epoxy resin curing [20]. Therefore, a combined imidazole/tertiary amine/alkanolamine accelerator system was selected in this work. However, the specific molar ratio of 2E4MI:DY070:DMP-30:TIPA = 9:1:18:2 was not directly adopted from the literature, but was determined through formulation screening. A series of accelerator ratios were evaluated using 2024-T3 aluminum bonded joints prepared from substrates supplied by Goodfellow Cambridge Ltd. (Huntingdon, UK) and cured at 85 °C for 6 h un-der 0.1 MPa. As shown in Table 1, the 9:1:18:2 formulation exhibited the highest lap shear strengths at both 25 °C and 100 °C, reaching 27.30 MPa and 22.70 MPa, respectively. Therefore, this accelerator formulation was selected as the optimized composition within the investigated composition range.

#### 2.2.2. Curing Process of Epoxy/Dicyandiamide Adhesive Film

The curing temperatures for the epoxy/dicyandiamide adhesive film were set to 65 °C, 75 °C, and 85 °C, with curing durations of 6 h and 24 h. The adhesion strength of the cured samples was evaluated for the determination of the optimal curing process. Simultaneously, the chemical structure, glass transition temperature, and decomposition behavior of the cured adhesive film were characterized via Fourier transform infrared spectroscopy (FTIR), dynamic mechanical analysis (DMA), and TGA.

### 2.3. Testing and Characterization

#### 2.3.1. Fourier Transform Infrared Spectroscopy (FTIR)

An IR Tracer-100 Fourier transform infrared spectrometer (Shimadzu, Kyoto, Japan) was utilized, operating over a scanning range of 450–4000 cm^−1^ with a resolution of 4 cm^−1^ and a scan rate of 32 scans/min.

#### 2.3.2. Dynamic Mechanical Analysis (DMA)

A Q400 dynamic mechanical analyzer (TA Instruments, New Castle, DE, USA) was utilized in a three-point bending mode with a vibration frequency of 1 Hz. The test temperature ranged from room temperature to 150 °C at a heating rate of 10 °C/min.

#### 2.3.3. Thermogravimetric Analysis (TGA)

A TGA55 thermogravimetric analyzer (TA Instruments, USA) was utilized for the evaluation of the decomposition behavior of the cured epoxy/dicyandiamide adhesive samples, operating under a nitrogen atmosphere over a temperature range of 25–800 °C with a heating rate of 10 °C/min.

#### 2.3.4. Mechanical Property Testing

An INSTRON-5696 universal testing machine (Instron, Norwood, MA, USA) was utilized to evaluate the mechanical properties of the bonded joints, including the lap shear strength of the Al-Al bonded joints, the bonding strength of the PMMA-PETR flexible bonded joints, and the 90° peel strength. For all the bonded joints, the nominal bondline thickness was controlled at 0.3 mm. The optimized bonded joints were cured at 85 °C for 6 h under 0.1 MPa. Before mechanical testing, all the bonded specimens were conditioned at 23 ± 2 °C and 40–60% relative humidity for 48 h. For the hydrothermally aged specimens, the samples were also equilibrated at 23 ± 2 °C and 40–60% relative humidity for 48 h before testing.

**Figure 3:**
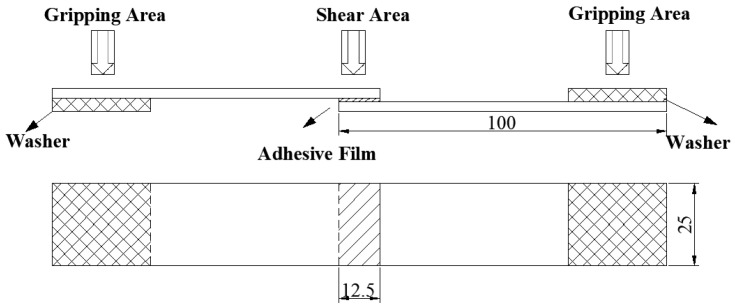
The shape and size of the Al-Al tensile lap shear specimens bonded with the epoxy/dicyandiamide adhesive. All dimensions are in mm, and the adhesive film thickness was 0.3 mm.

Shear Strength: Both the Al-Al and PMMA-PETR substrates were employed for the lap shear strength evaluation of the epoxy/dicyandiamide/composite accelerator adhesive film. The 2024-T3 aluminum alloy substrates possessed dimensions of 100 mm × 25 mm × 3 mm, with a bonding area of 25 mm × 12.5 mm, corresponding to 312.5 mm^2^. The aluminum substrates used for the Al-Al bonded joints were 2024-T3 aerospace aluminum alloy. The bonding surfaces of these substrates were initially wiped with acetone for oil removal, subsequently abraded with 120-mesh sandpaper, and finally cleansed with acetone. The bonded specimen of the Al substrate is schematically illustrated in Figure 3. The Al-Al bonded joints were utilized for the characterization of the baseline lap shear strength of the adhesive film in accordance with GB/T 7124-2008 [21], Adhesives-Determination of tensile lap shear strength of rigid-to-rigid bonded assemblies, which is identical to ISO 4587:2003 [22].

**Figure 4:**
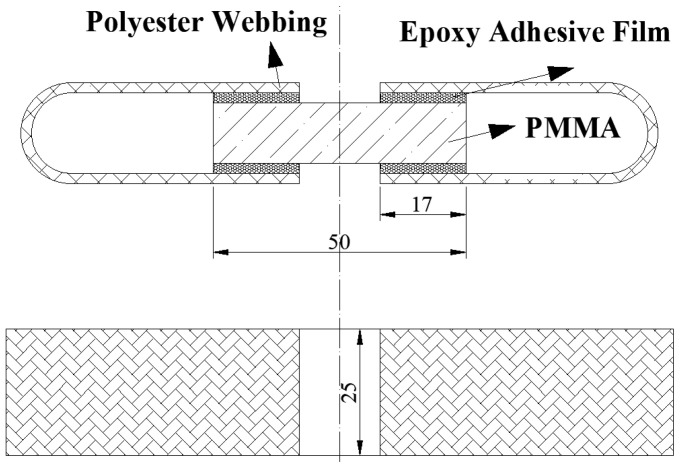
The shape and size of the PMMA-PETR flexible joint specimens bonded with the epoxy/dicyandiamide adhesive film. All dimensions are in mm, and the adhesive film thickness was 0.3 mm.

The bonded joint consisting of the PMMA sheet and the PETR was fabricated for the simulation of the joint structure at the cockpit flexible connection. Therein, the PMMA samples possessed dimensions of 9 mm × 25 mm × 50 mm, with a specimen width of 25 mm and a single-side bonding length of 17 mm between the PMMA and PETR. The bonding area of the PMMA was initially cleaned with 120 #solvent oil, a petroleum-based cleaning solvent, followed by surface polishing with sandpaper and final cleaning. The PETR was heat-treated at 195 °C for 30 min and then cleaned with analytical-grade acetone. The bonded PMMA-PETR specimen is shown in Figure 4. The specimens were evaluated for lap shear strength at room temperature and 100 °C, respectively, at a testing rate of 10 mm/min. According to HB 5186-2011 (Specification for adhesive used in flexible joint of aircraft transparency) [23], the bonding strength of the PMMA-PETR flexible bonded joint was calculated by dividing the maximum failure load by the specimen width of 25 mm.

90° Peel Strength: Both the Al-Al and PMMA-PETR substrates were also employed for the 90° peel strength evaluation of the epoxy/dicyandiamide/composite accelerator adhesive film. The Al substrates possessed dimensions of 3 mm × 20 mm × 200 mm and were subjected to the same surface preparation procedure described above. The PMMA-PETR specimens, utilized for the evaluation of the adhesion performance of the adhesive film toward the cockpit flexible connection structure, possessed dimensions of 9 mm × 20 mm × 200 mm and were treated identically. An actual bonding width of 20 mm and a bonding length of 150 mm were applied to both types of specimens, wherein an unbonded free end of 50 mm was reserved for gripping by the testing machine fixtures. The 90° peel strengths of both the Al-Al and PMMA-PETR bonded joints at room temperature and 100 °C were evaluated at a testing rate of 100 mm/min in accordance with the Chinese Military Standard GJB 446-88, Test method for 90° peel strength of adhesives (metal-to-metal) [24]. For both the Al-Al and PMMA-PETR 90° peel specimens, the actual bonding width was 20 mm and the bonding length was 150 mm. The 90° peel strength was calculated by dividing the average peel load during the stable peeling stage by the specimen width of 20 mm, and the results were expressed in N/cm.

Shear strength formula of bonded joints,

(1) $$A=\frac{P}{S}$$

where *A* is the tensile lap shear strength (MPa), P is the maximum failure load (N), and S is the bonding area, namely 312.5 mm^2^. The arithmetic average of 5 samples was adopted for the final result.

Shear strength formula of PMMA-PETR flexible bonded joints

(2) $$A=\frac{P}{B}\times 10^{3}$$

where A is the shear strength of the PMMA-PETR flexible bonded joint (kN/m), P is the maximum failure load (kN), and B is the average specimen width, namely 25 mm. The factor of 10^3^ is used to convert kN/mm to kN/m. The arithmetic average of 5 samples was adopted for the final result.

### 2.4. Hydrothermal Aging Test

The PMMA-AF-PETR structures were exposed to a hydrothermal aging environment within a climate chamber under conditions of 80 °C and 90% relative humidity (RH) for a total duration of 600 h. Therein, the specimens were retrieved at intervals of 20 h during the initial 100 h of exposure, and at intervals of 100 h thereafter. Prior to the comprehensive evaluations—including FTIR, TGA, shear strength (at 100 °C), and 90° peel strength (at 100 °C)—the specimens were equilibrated at a temperature of 23 °C ± 2 °C and a relative humidity of 50% ± 10% for 48 h. Specifically, the service temperature of the PMMA-AF-PETR structure could reach 100 °C under special operating conditions during actual service. Therefore, the evaluation of the bonding strength of the PMMA-AF-PETR structure was conducted after hydrothermal aging under 100 °C.

## 3. Results and Discussion

### 3.1. Impact of Curing Processes on the Structure of Epoxy/Dicyandiamide Adhesive Films

Processing conditions involving a curing temperature of ≤85 °C and a curing duration of <8 h were primarily verified for compliance with the curing process constraints imposed by the cockpit edge flexible connection on bonding materials. Simultaneously, the structure–property evolutions of the epoxy/dicyandiamide adhesive film modified with the imidazole–alkanolamine composite accelerator were characterized via Fourier transform infrared spectroscopy (FTIR), a universal testing machine, dynamic mechanical analysis (DMA), and TGA.

#### 3.1.1. Adhesion Performance Analysis Under Different Curing Processes

The comprehensive mechanical properties of the Al-Al and PMMA-PETR bonded joints were evaluated using the adhesive film, wherein the specimen preparation procedure and the testing results are presented in Section 2.3.4 and Table 2, respectively. Complete curing of the adhesive film modified with the composite accelerator was achieved within a temperature range of 65 °C to 85 °C. Following curing at 85 °C for 6 h, a room-temperature lap shear strength of 27.30 MPa (satisfying the design criterion of ≥25.00 MPa), a room-temperature 90° peel strength of 39.0 N/cm (satisfying the design criterion of ≥35.0 N/cm), and a 100 °C lap shear strength of 22.70 MPa (satisfying the design criterion of ≥15.00 MPa) were exhibited by the Al-Al lap joints. Meanwhile, for the PMMA-AF-PETR bonded joints, a room-temperature bonding strength of 243.52 kN/m (satisfying the design criterion of ≥200.00 kN/m), a room-temperature 90° peel strength of 42.2 N/cm (satisfying the design criterion of ≥23.0 N/cm), a 100 °C bonding strength of 203.62 kN/m (satisfying the design criterion of ≥100.00 kN/m), and a 100 °C 90° peel strength of 38.6 N/cm (satisfying the design criterion of ≥23.0 N/cm) were yielded, whereby the design requirements for the comprehensive mechanical properties were successfully fulfilled. Furthermore, a reduction to zero in the bonding strength at 100 °C was observed for the PMMA-AF-PETR structures cured at 65 °C for 6 h. This phenomenon is attributed to the low initial crosslinking density of the system under this condition, whereby the glass transition temperature (Tg) of the adhesive layer was exceeded at 100 °C, resulting in structural softening and a subsequent loss of cohesive strength. It is demonstrated through the investigation that a significant effect on the reduction in the curing temperature of the epoxy/dicyandiamide system is exerted by the imidazole–alkanolamine composite accelerator, and a superior performance matching is manifested with the curing process of 85 °C for 6 h.

#### 3.1.2. Structural Analysis Under Different Curing Processes

**Figure 5:**
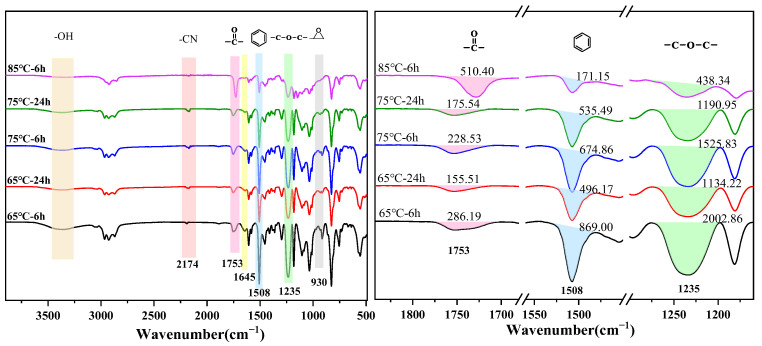
The FTIR of curing processes on structure of cured samples.

**Figure 6:**
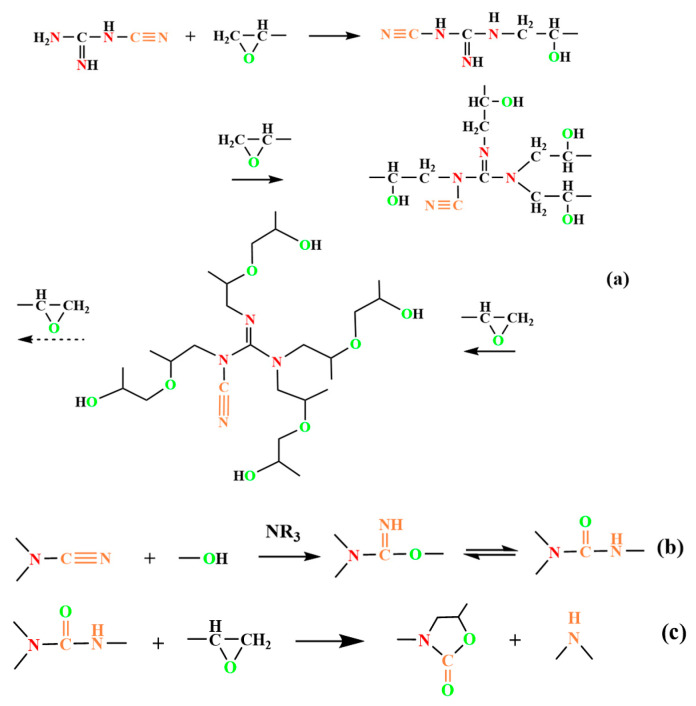
Proposed curing reaction mechanism of the epoxy/dicyandiamide adhesive [1,16]: (**a**) epoxy ring-opening reaction of dicyandiamide and subsequent crosslinked-network formation promoted by the composite accelerator; (**b**) reac-tion between cyano groups and hydroxyl groups; (**c**) reaction between urea groups and epoxy groups to form oxa-zolidinone structures.

FTIR was employed to characterize the structural changes within the curing samples to evaluate the effects of different curing processes, and the results are illustrated in Figure 5. In the FTIR spectra of the epoxy/dicyandiamide cured system, the characteristic absorption peaks are located at 930 cm^−1^ for terminal epoxy groups, 2174 cm^−1^ for C≡N (cyano) groups, 3300–3400 cm^−1^ for -OH (hydroxyl) groups, 1753 cm^−1^ for the carbonyl group in oxazolidinone [14], 1645 cm^−1^ for C=N (imine) groups, 1235 cm^−1^ for -C-O-C- (ether) bonds, and 1508 cm^−1^ for the benzene ring. This benzene ring structure remained essentially unchanged during the curing process and was utilized as a reference peak intensity to calibrate the variations in other functional groups, with the integration calculations of the primary peaks presented in Table 3. In Table 3, A represents the oxazolidinone carbonyl peak area at 1753 cm^−1^, B represents the benzene ring peak area at 1508 cm^−1^, and C represents the ether peak area at 1235 cm^−1^. A/B and C/B denote the corresponding peak area ratios. Different curing processes exerted significant effects on the structure of the epoxy/dicyandiamide cured samples (with the absorption peak intensity of the benzene ring at 1508 cm^−1^ selected as the calibration reference). Specifically, for the cured samples obtained at 85 °C for 6 h, the characteristic absorption peaks of the terminal epoxy groups at 930 cm^−1^, cyano groups at 2174 cm^−1^, and hydroxyl groups at 3300–3500 cm^−1^ disappeared, while the characteristic absorption peak of the imine groups at 1645 cm^−1^ vanished almost completely. Concurrently, both the oxazolidinone characteristic absorption peak at 1753 cm^−1^ and the ether bond characteristic absorption peak at 1235 cm^−1^ were significantly enhanced (Table 3), which indicated that deep crosslinking was achieved between the epoxy groups and active centers within the system, such as cyano groups, intermediate hydroxyl groups, and urea groups (as shown in Figure 6). In conjunction with the optimal mechanical properties presented in Table 2, an increase in the rigid crosslinking density and an enhancement in the cohesive strength of the material were achieved as the curing reaction proceeded. Furthermore, the elevated content of flexible segments such as ether bonds served to effectively dissipate internal stress and suppress the occurrence of brittle fracture, which ultimately significantly improved the failure limit of the material.

#### 3.1.3. Thermomechanical and Thermal Decomposition Behavior Analysis

**Figure 7:**
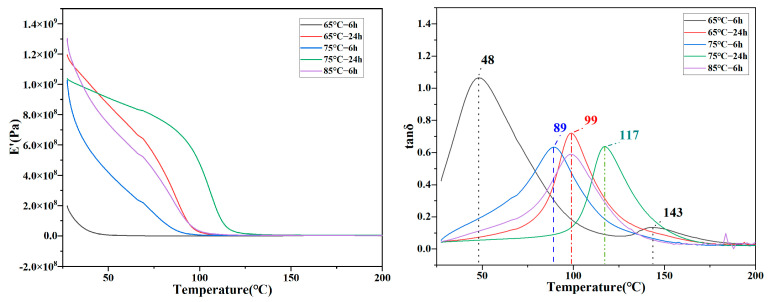
The effects of cured processes on dynamic mechanical behavior of cured samples.

The glass transition temperature (Tg) is a critical thermal property indicator for cured epoxy/dicyandiamide adhesive film samples. Physically, it represents the minimum temperature required for molecular segments to transition from a frozen state to a cooperative motion realized via the internal rotation of main-chain single bonds, which provides crucial guidance for the practical application of adhesive films. In this study, DMA was conducted on the cured samples obtained from different curing processes, wherein the temperature corresponding to the maximum value of the loss factor (tan *δ*) in the curves was defined as the Tg of the specimens, with the results illustrated in Figure 7. DMA analysis indicated that, except for the 65 °C/6 h cured sample, a single distinct peak was exhibited in the tan *δ* curves of the remaining samples, demonstrating a concentrated glass transition process. The Tg values of the cured samples obtained at 65 °C/6 h, 65 °C/24 h, 75 °C/6 h, 75 °C/24 h, and 85 °C/6 h were 48 °C, 99 °C, 89 °C, 117 °C, and 99 °C, respectively. These results demonstrated that both an increase in the curing temperature and an extension of the curing duration could elevate the Tg of the cured samples in the presence of active crosslinking sites, with the effect of the latter being more pronounced. Although the 85 °C/6 h sample exhibited a lower Tg, it demonstrated excellent high-temperature adhesion performance. This phenomenon was potentially attributed to the more comprehensive reaction of active sites—such as cyano, epoxy, and hydroxyl groups—under this specific curing process, which led to increased contents of oxazolidinone structures and ether bonds within the cured samples (as Table 3 shown).

Although the formation of oxazolidinone structures contributed to the enhancement in chain rigidity, the attenuation of hydrogen bonding interactions induced by hydroxyl consumption and the “softening effect” brought about by the increased ether bonds played a dominant role. This structural evolution served to enhance the interactions at the bonding interface, ultimately resulting in a decreased Tg accompanied by an elevated adhesion strength. Additionally, due to a lower initial crosslinking density, certain functional groups with high activation energies in the 65 °C/6 h sample failed to react comprehensively and underwent further crosslinking during the heating process. Consequently, the sample exhibited an anomalous phenomenon characterized by an initial Tg of 48 °C and a peak Tg of 143 °C, which indicated the presence of significant post-curing behavior and severe phase separation, thereby rendering its Tg data of limited reference value.

**Figure 8:**
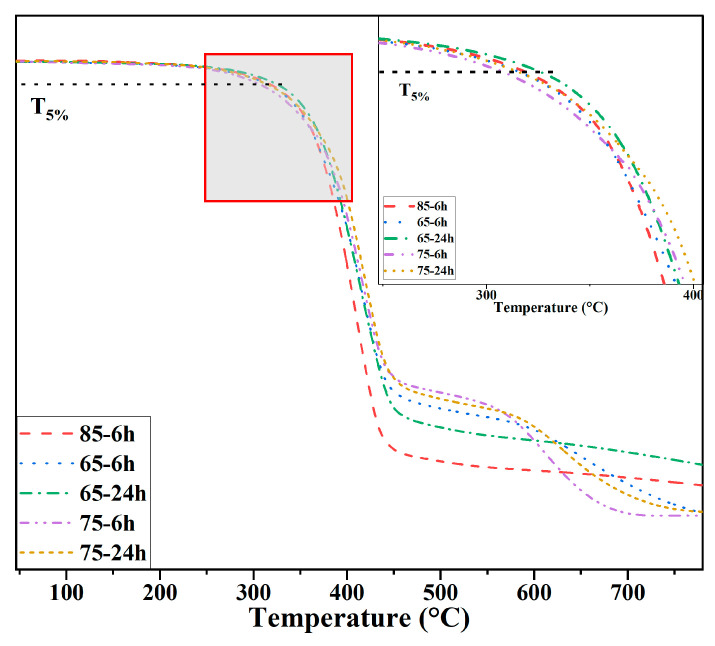
The effects of cured processes on thermal degradation of cured samples.

Thermogravimetric Analysis (TGA): TGA was performed to characterize the thermal decomposition behavior of the cured epoxy/dicyandiamide adhesive films obtained under different curing processes. The corresponding TGA curves are shown in Figure 8, and the characteristic temperatures, including the 5% weight loss temperature (T_5%_) and the peak weight loss temperature (T_max_), are listed in Table 4.

As shown in Table 4, the T_5%_ values of the cured samples ranged from 308 °C to 326 °C, while the T_max_ values remained within 406–416 °C. These results indicate that the main thermal decomposition process of the cured adhesive films occurred above 400 °C under a dynamic nitrogen atmosphere. Among the investigated curing processes, the adhesive film cured at 85 °C for 6 h exhibited a T_5%_ of 319 °C and a T_max_ of 416 °C. Therefore, the TGA results describe the thermal decomposition behavior of the cured adhesive films under programmed heating conditions, rather than general long-term thermal resistance.

### 3.2. Hydrothermal Resistance of Epoxy/Dicyandiamide Adhesive Film

#### 3.2.1. Adhesion Performance Analysis After Hydrothermal Aging

The adhesive film was cured at 85 °C for 6 h, followed by hydrothermal aging testing under environmental conditions of 80 °C and 90% relative humidity (RH) with a total specified exposure duration of 600 h. Intensive sampling was conducted at intervals of 20 h within the 0–100 h window to capture the performance variations during the early stage of aging. Thereafter, sample collection and adhesion performance evaluations were performed at 100 h intervals, with the results summarized in Table 5 and Table 6. Given the substantial decline in the strength retention of the samples during the 0–100 h exposure window, four additional sampling points at 20, 40, 60, and 80 h were incorporated within this early-stage aging cycle to ensure the reliability of the evaluation results. The corresponding testing data are presented in Table 7 and Table 8.

As listed in Table 5, Table 6, Table 7 and Table 8, hydrothermal aging exerts a significant effect on the PMMA-AF-PETR structure. With the increasing aging time, the strength of the bonded samples exhibited a marked decrease followed by a stabilization trend. Specifically, after 20 h of hydrothermal aging, reductions of 60.73% and 73.09% were observed in the lap shear strength and 90° peel strength of the samples, respectively. Upon reaching 100 h of exposure, the mechanical properties tend to stabilize, with ultimate strength reductions of approximately 75% and 80%.

The rapid degradation of the adhesion strength is primarily attributed to three aspects. Firstly, the formation of hydrogen bonds between the oxygen/nitrogen atoms in the epoxy/dicyandiamide system and water molecules triggers moisture expansion and plasticization effects, which leads to network plasticization, segment relaxation, and the subsequent deterioration in the mechanical performance [25]. Secondly, the hydrolysis of polar hydrophilic groups in the epoxy resin is induced under the synergistic hydrothermal effect, which reduces the internal crosslinking density and causes a rapid reduction in the mechanical properties. Thirdly, the bonded PETR is a woven fiber material with high hygroscopicity, allowing the hydrothermal effects of the aging environment to continuously act on the bonding interface; concurrently, active small-molecule chain segments generated via aging degradation can be continuously leached out by the high-temperature water, further accelerating interfacial failure.

#### 3.2.2. Structural Analysis After Hydrothermal Aging

**Figure 9:**
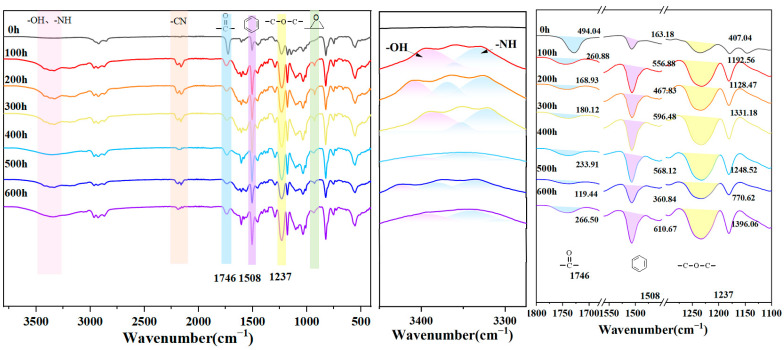
The FTIR curves of 0–600 h hydrothermally aged specimens.

**Figure 10:**
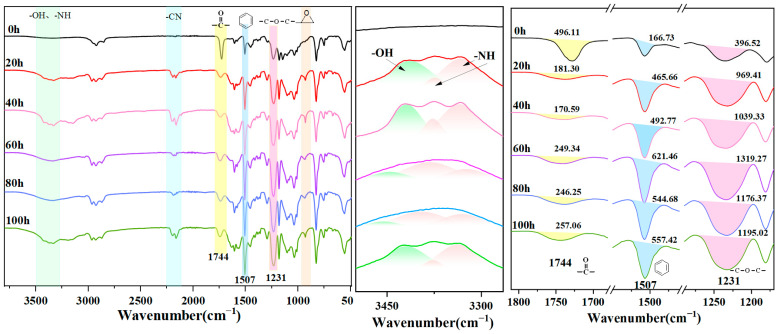
The FTIR curves of 0–100 h hydrothermally aged specimens.

FTIR spectroscopy was conducted on the epoxy/dicyandiamide adhesive films subjected to various hydrothermal aging times to investigate the chemical structural evolutions during the aging process and to provide auxiliary insights into the macroscopic mechanical property variations. The results are shown in Figure 9 and Figure 10, and Table 9. In Table 9, A represents the oxazolidinone carbonyl peak area at 1753 cm^−1^, B represents the benzene ring peak area at 1508 cm^−1^, and C represents the ether peak area at 1235 cm^−1^. A/B and C/B denote the corresponding peak area ratios.

As illustrated in Figure 9 and Figure 10, the oxazolidinone carbonyl absorption peak at 1753 cm^−1^ weakens following hydrothermal aging, concurrently with the emergence of characteristic cyano peaks at 2162 cm^−1^ and 2197 cm^−1^ and irregular absorption bands within the 3300–3500 cm^−1^ region. Peak deconvolution fitting showed variations in hydroxyl- and amino-related absorption bands, which may be associated with moisture-induced plasticization, partial hydrolysis, and physical disruption of the crosslinked network during hydrothermal aging.

**Figure 11:**
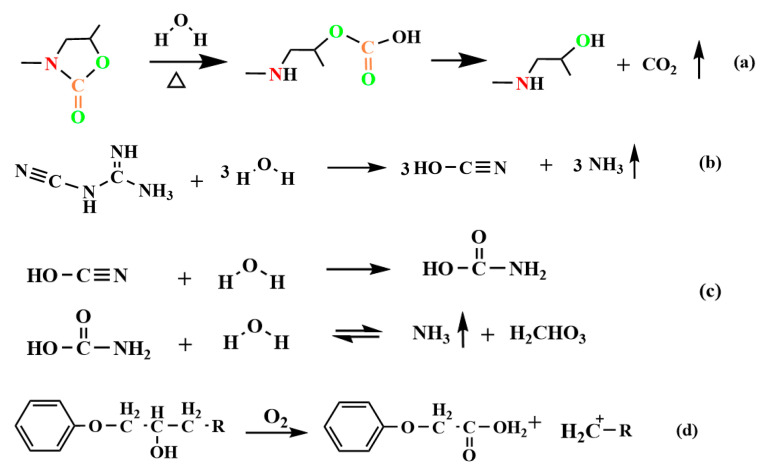
Schematic illustration of the possible hydrothermal degradation pathway. (**a**) hydrolysis and decarboxylation of oxazolidinone structures; (**b**) hydrolysis of dicyandiamide-derived structures; (**c**) hydrolysis of cyanamide/carbamic acid intermediates with the formation of NH_3_ and H_2_CO_3_; (**d**) oxidative degradation of ether-containing epoxy seg-ments.

Based on these FTIR changes and the corresponding decrease in adhesion performance, a possible hydrothermal degradation pathway is proposed in Figure 11. During the hydrothermal aging process, water molecules may diffuse into the resin matrix and interact with polar oxygen- and nitrogen-containing groups in the epoxy/dicyandiamide network. This process may weaken intermolecular interactions, increase the mobility of molecular segments, and induce plasticization of the adhesive layer. Meanwhile, under the combined effect of heat and moisture, unstable structures such as oxazolidinone rings and incompletely reacted dicyandiamide-related structures may undergo partial ring opening, hydrolysis, or structural rearrangement, as schematically shown in Figure 11a–c. These reactions may contribute to the formation of hydroxyl-, amino-, carbonyl-, or cyano-related species observed in the FTIR spectra. In addition, oxygen diffusion during aging may cause oxidative changes in the adhesive layer, as illustrated in Figure 11d. The possible formation or leaching of low-molecular-weight species, together with plasticization and weakening of the crosslinked network, may account for the decrease in bonding strength and 90° peel strength after hydrothermal aging, which is consistent with the mechanical data in Table 5, Table 6, Table 7 and Table 8.

#### 3.2.3. Thermogravimetric Analysis After Hydrothermal Aging

**Figure 12:**
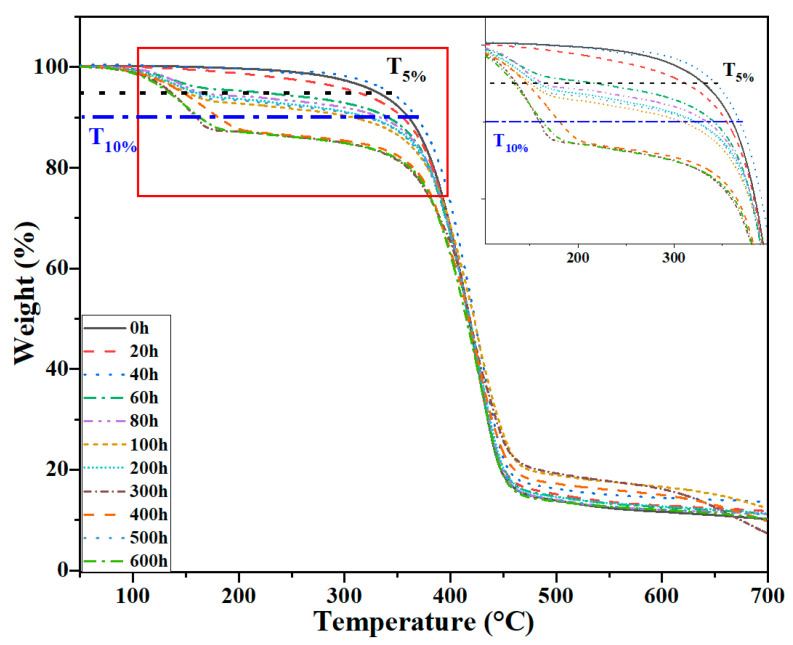
TGA curves of hydrothermally aged specimens.

TGA was performed to evaluate the influence of hydrothermal aging on the mass loss behavior of the adhesive film under dynamic heating conditions. The TGA curves are shown in Figure 12, and the corresponding thermogravimetric characteristic parameters are listed in Table 10. The low-temperature mass loss may be partly associated with absorbed moisture introduced during hydrothermal aging and volatile low-molecular-weight species formed or released during the aging process. In contrast, the peak weight loss temperature remained within 414–420 °C, indicating that the main high-temperature decomposition process of the bulk adhesive network was not markedly changed after hydrothermal aging. Therefore, the TGA results mainly reflect the influence of hydrothermal aging on the mass loss behavior of the adhesive film during thermal analysis, rather than a general evaluation of long-term thermal stability.

## 4. Conclusions

The imidazole/alkanolamine composite accelerator enabled the epoxy/dicyandiamide adhesive film to be cured at 85 °C. After curing at 85 °C for 6 h, the Al-Al bonded joints exhibited lap shear strengths of 27.30 MPa at room temperature and 22.70 MPa at 100 °C, together with 90° peel strengths of 39.0 N/cm and 39.7 N/cm, respectively. For the PMMA-PETR flexible bonded joints, the bonding strengths reached 243.52 kN/m at room temperature and 203.62 kN/m at 100 °C, while the corresponding 90° peel strengths were 42.2 N/cm and 38.6 N/cm, respectively. These values satisfied the design requirements for cockpit edge flexible bonding. Therefore, the developed one-component epoxy/dicyandiamide adhesive film is suitable for the target application scenario where low-temperature curing, convenient processing, and controlled assembly of PMMA-PETR flexible joints are required. However, hydrothermal aging caused a pronounced decrease in the adhesion performance of the PMMA-AF-PETR bonded structure, indicating that moisture ingress can damage the bonded interface in the absence of sufficient protection. These results indicate that moisture and oxygen ingress can markedly weaken the PMMA-AF-PETR bonded structure under hygrothermal conditions; therefore, external edge sealing or sealed assembly is necessary to maintain long-term service stability. In practical applications, compatible polysulfide, polyurethane, or silicone sealants, as well as sealing tapes, may be used, and the service life, inspection intervals, and maintenance requirements should be determined according to the actual sealing design and service environment.

## Figures and Tables

**Table 1:** Effect of 2E4MI:DY070:DMP-30:TIPA ratio on the shear strength of Al-Al bonded joints.

2E4MI:DY070:DMP-30:TIPA	9:1:25:09:1:25:0	9:1:20:0	9:1:19:1	9:1:18:2	9:1:17:3
25 °C shear strength, MPa	10.13	16.87	22.19	27.30	21.97
100 °C shear strength, MPa	0	6.24	9.33	22.70	12.64

Subsequently, the kneaded epoxy resin–dicyandiamide composite accelerator mixture was transferred into a film casting machine, wherein the epoxy adhesive film was fabricated through film formation and winding processes, followed by storage at −18 °C for future use.

**Table 2:** Effects of different curing processes on the mechanical properties of Al-Al and PMMA-PETR bonded joints.

Test Temperature/°C	Curing Temperature/°C	Curing Time/h	Al-Al				PMMA-PETR			
			Shear Strength (Designed) /MPa	90° Peel Strength (Designed)/(N/cm)	Shear Strength (Tested) /MPa	90° Peel Strength (Tested) /(N/cm)	Shear Strength (Designed)/ (kN/m)	90° Peel Strength (Designed) /(N/cm)	Shear Strength (Tested)/ (kN/m)	90° Peel Strength (Tested)/ (N/cm)
25	65	6	25	35	6.45 ± 0.68	7.7 ± 1.2	200	23	126.21 ± 1.76	15.3 ± 1.2
		24			14.65 ± 0.13	9.5 ± 1.3			131.69 ± 2.67	22 ± 1.6
	75	6			16.31 ± 0.36	14.4 ± 0.6			157.65 ± 3.51	18.7 ± 1.6
		24			15.19 ± 0.72	14.5 ± 0.7			170.54 ± 2.92	26 ± 2.9
	85	6			27.30 ± 0.94	39.0 ± 2.4			243.52 ± 6.91	42.2 ± 3.2
100	65	6	15	-	1.00 ± 0.09	40.5 ± 2.3	100	23	0.00	12.4 ± 0.6
		24			12.4 ± 0.91	38.5 ± 2.7			52.44 ± 6.98	18.3 ± 1.2
	75	6			9.31 ± 0.47	32.1 ± 2.0			49.64 ± 4.87	22.1 ± 2
		24			13.51 ± 0.27	39.8 ± 2.0			137.16 ± 2.95	25.4 ± 0.11
	85	6			22.70 ± 0.86	39.7 ± 2.2			203.62 ± 7.94	38.6 ± 4.5

**Table 3:** The effects of curing processes on the infrared absorption peaks of cured samples.

Condition	A	B	C	A/B	C/B
85 °C-6 h	510.40	171.15	438.34	2.98	2.56
75 °C-24 h	175.54	535.49	1190.95	0.33	2.22
75 °C-6 h	228.53	674.86	1525.83	0.34	2.26
65 °C-24 h	155.51	496.17	1134.22	0.31	2.29
65 °C-6 h	286.19	869.00	2002.86	0.33	2.30

A represents the oxazolidinone carbonyl peak area at 1753 cm^−1^, B represents the benzene ring peak area at 1508 cm^−1^, and C represents the ether peak area at 1235 cm^−1^; A/B and C/B denote the peak area ratios.

**Table 4:** TGA data of different curing processes.

Curing Process	T_5%_/°C	T_max_/°C
65 °C/6 h	316	406
65 °C/24 h	326	407
75 °C/6 h	308	410
75 °C/24 h	319	414
85 °C/6 h	319	416

**Table 5:** Shear strength of 0–600 h hydrothermally aged specimens at 100 °C.

Aging Time/h	0	100	200	300	400	500	600
Shear strength, kN/m	185.08 ± 9.72	41.92 ± 7.40	41.81 ± 4.91	50.13 ± 9.99	64.855 ± 4.65	49.58 ± 5.91	49.85 ± 13.44
Strength retention rate, %	100.00	22.65	22.59	27.08	35.03	26.78	26.93

**Table 6:** Peel strength of 0–600 h hydrothermally aged specimens at 100 °C.

Aging Time/h	0	100	200	300	400	500	600
Peel strength, N/cm	48.3 ± 3.1	8.4 ± 0.5	7 ± 0.8	4.3 ± 1.8	8.6 ± 0.6	7.1 ± 1.5	7.9 ± 0.9
Strength retention rate, %	100.00	17.39	14.49	8.90	17.80	14.69	16.35

**Table 7:** Shear strength of 0–100 h hydrothermally aged specimens at 100 °C.

Aging Time/h	0	20	40	60	80	100
Shear strength, kN/m	185.08 ± 10.19	72.69 ± 9.24	59.63 ± 3.70	61.01 ± 7.60	81.16 ± 4.74	41.92 ± 7.40
Strength retention rate, %	100.00	39.27	32.21	32.96	43.85	22.65

**Table 8:** Peel strength of 0–100 h hydrothermally aged specimens at 100 °C.

Aging Time/h	0	20	40	60	80	100
Peel strength, N/cm	48.3 ± 3.3	13 ± 7.4	9 ± 1.2	9.2 ± 0.8	10.4 ± 2.7	8.4 ± 0.5
Strength retention rate, %	100.00	26.91	18.63	19.04	21.53	17.39

**Table 9:** The effects of hydrothermal aging on the infrared absorption peaks of cured samples.

Condition	A	B	C	A/B	C/B
0 h	496.11	166.73	396.52	2.98	2.38
20 h	181.30	465.66	969.41	0.39	2.08
40 h	170.59	492.77	1039.33	0.35	2.11
60 h	249.34	621.46	1319.27	0.40	2.12
80 h	246.25	544.68	1176.37	0.45	2.16
100 h	257.06	557.42	1195.02	0.46	2.14
200 h	168.93	467.83	1128.47	0.36	2.41
300 h	180.12	596.48	1331.18	0.30	2.23
400 h	233.91	568.12	1248.52	0.41	2.20
500 h	119.44	360.84	770.62	0.33	2.14
600 h	266.50	610.67	1396.06	0.44	2.29

A represents the oxazolidinone carbonyl peak area at 1753 cm^−1^, B represents the benzene ring peak area at 1508 cm^−1^, and C represents the ether peak area at 1235 cm^−1^; A/B and C/B denote the peak area ratios.

**Table 10:** Thermogravimetric results.

Hydrothermal Aging Time/h	5%Weight Loss Temperature/°C	10%Weight Loss Temperature/°C	Peak Weight Loss Temperature/°C	Peak Weight Loss Ratio/%
0	331	361	414	54.05
20	312	354	414	53.12
40	343	368	419	54.41
60	218	341	415	52.22
80	163	334	417	51.57
100	150	308	420	52.35
200	158	323	417	51.50
300	136	160	419	49.18
400	146	181	418	49.29
500	155	320	418	50.07
600	133	163	418	47.28

## Data Availability

The data presented in this study are available on request from the corresponding author due to commercial confidentiality restrictions related to the potential industrial application of the adhesive film.

## References

[1] Güthner T., Hammer B. (1993). Curing of epoxy resins with dicyandiamide and urones. J. Appl. Polym. Sci..

[2] Yang H., Zhang Y., Zhao X., Huang Z. (2018). Research progress on toughening modification of epoxy resin. Eng. Plast. Appl..

[3] Turon A., Camanho P.P., Costa J., Dávila C.G. (2006). A damage model for the simulation of delamination in advanced composites under variable-mode loading. Mech. Mater..

[4] Ham Y.R., Kim S.H., Shin Y.J., Lee D.H., Yang M., Min J.H., Shin J.S. (2010). A comparison of some imidazoles in the curing of epoxy resin. J. Ind. Eng. Chem..

[5] Ooi S.K., Cook W.D., Simon G.P., Such C.H. (2000). DSC studies of the curing mechanisms and kinetics of DGEBA using imidazole curing agents. Polymer.

[6] Hesabi M., Salimi A., Beheshty M.H. (2017). Effect of tertiary amine accelerators with different substituents on curing kinetics and reactivity of epoxy/dicyandiamide system. Polym. Test..

[7] Liu X.D., Sudo A., Endo T. (2011). Efficient accelerating effect of carbonyldiimidazole on epoxy-dicyandiamide curing system. J. Polym. Sci. Part A Polym. Chem..

[8] Smith I.T. (1961). The mechanism of the crosslinking of epoxide resins by amines. Polymer.

[9] Stammen E., Bergenthun F., Brokamp S., Dilger K. (2025). Design of bio-based adhesives for fuel cell applications. J. Adhes..

[10] Kablov E.N., Startsev V.O. (2020). Climatic aging of aviation polymer composite materials: II. Development of methods for studying the early stages of aging. Russ. Metall. (Met.).

[11] Kablov E.N., Startsev O.V., Krotov A.S., Kirillov V.N. (2011). Climatic aging of composite aviation materials: II. Relaxation of the initial structural nonequilibrium and through-thickness gradient of properties. Russ. Metall. (Met.).

[12] Kablov E.N., Startsev O.V., Krotov A.S., Kirillov V.N. (2012). Climatic aging of composite aviation materials: III. Significant aging factors. Russ. Metall. (Met.).

[13] Roylance D., Roylance M. (1978). Weathering of Fiber-reinforced Epoxy Composites. Polym. Eng. Sci..

[14] Afshar A., Alkhader M., Korach C.S., Chiang F.P. (2015). Effect of long-term exposure to marine environments on the flexural properties of carbon fiber vinylester composites. Compos. Struct..

[15] Sousa J.M., Correia J.R., Cabral-Fonseca S. (2016). Durability of glass fibre reinforced polymer pultruded profiles: Comparison between QUV accelerated exposure and natural weathering in a Mediterranean climate. Exp. Tech..

[16] Startsev O.V., Mashinskaya G.P., Yartsev V.A. (1985). Molecular mobility and relaxation processes in an epoxy matrix 2. Effects of weathering in humid subtropical climate. Mech. Compos. Mater..

[17] Saunders T.F., Levy M.F., Serino J.F. (1967). Mechanism of the tertiary amineâ catalyzed dicyandiamide cure of epoxy resins. J. Polym. Sci. Part A1 Polym. Chem..

[18] Padma A., Rao R.M.V.G.K., Subramaniam C., Nagendrappa G. (1995). Cure characterization of triglycidyl epoxy/aromatic amine systems. J. Appl. Polym. Sci..

[19] Yang T., Zhang C., Zhang J., Cheng J. (2014). The influence of tertiary amine accelerators on the curing behaviors of epoxy/anhydride systems. Thermochim. Acta.

[20] Liu S., Liu Z., Jiang D., Zong R., Zhong A., Shentu B. (2025). Synergistic effects of tertiary amine and imidazole accelerators on epoxy resin curing. J. Appl. Polym. Sci..

[21] (2008). Adhesives-Determination of Tensile Lap-Shear Strength of Rigid-to-Rigid Bonded Assemblies.

[22] (2003). Adhesives-Determination of Tensile Lap-Shear Strength of Rigid-to-Rigid Bonded Assemblies.

[23] (2011). Specification for Adhesive Used in Flexible Ioint of Aircraft Transparency.

[24] (1988). Test Method for 90° Peel Strength of Adhesives (Metal to Metal).

[25] Viana G., Costa M., Banea M., da Silva L. (2016). A review on the temperature and moisture degradation of adhesive joints. Proc. Inst. Mech. Eng. Part L J. Mater. Des. Appl..

